# Spatio-temporal distribution and agroecological factors associated with canine leptospirosis in Great Britain

**DOI:** 10.1016/j.prevetmed.2021.105407

**Published:** 2021-08

**Authors:** C. Taylor, D.C. Brodbelt, B. Dobson, B. Catchpole, D.G. O’Neill, K.B. Stevens

**Affiliations:** a Royal Veterinary College, Hawkshead Lane, Hatfield, Hertfordshire, United Kingdom; b Imperial College London, Exhibition Rd, South Kensington, London, United Kingdom

**Keywords:** Agroecological, Disease distribution, Ecological niche modelling, Leptospirosis, MaxEnt, Spatial analysis

## Abstract

Leptospirosis is an important global zoonotic disease that affects a wide range of mammalian species. Canine leptospirosis outbreaks have been reported after metereological events such as flooding (eg. in Brazil and the United States of America) suggesting an environmental association, but there has been no such study in Great Britain (GB). The distribution of cases across GB is also unreported. Objectives of this study were to: (1) assess the spatio-temporal variation of leptospirosis test submissions (2) explore associations between agroecological risk factors and distribution of different canine leptospirosis serogroups in GB, and (3) generate probability of presence maps for the different serogroups. Data analysed comprised laboratory submissions (n = 3986) to IDEXX laboratories between 1^st^ January 2009 and 31^st^ December 2018 for PCR or MAT leptospirosis testing. Spatial and seasonal scan statistics were used to investigate spatial and temporal clustering of positive tests, logistic regression was used to identify significant agroecological risk factors for positive tests, and the Maxent algorithm was used to model the environmental niche of four serogroups. There was an increased risk of a positive test result in the West Midlands of England (relative risk = 2.16) and between October and January (relative risk = 1.54). Logistic regression identified season and region to be significantly associated with a positive diagnosis,with higher odds of a positive test in Autumn (OR = 1.86 95 %CI 1.29−2.69) and Winter (OR = 1.51, 95 %CI 1.02−2.23) and in the East (OR = 2.20, 95 %CI = 1.31−3.71) and West Midlands (OR = 2.32, 95 %CI 1.45−3.71). The increased test-positive proportion in Autumn together with the increased odds of a positive diagnosis in Autumn suggests there may be a seasonal pattern to the canine leptospirosis in GB. The most important variable associated with higher leptospirosis presence in all ecological niche models was higher average annual temperature. The importance and retention of other variables differed between serogroups. Overall, a higher probability of leptospirosis presence was predicted in southern England and a low probability in Scotland and northern England. Although leptospirosis vaccine usage provides protection against the majority of serogroups identified here, one is not represented in the currently licensed vaccine formulations and therefore leptospirosis should remain a differential diagnosis in vaccinated dogs demonstrating consistent clinical signs of the disease.

## 1 Introduction

Leptospirosis is an important zoonotic disease with a global distribution generally of greatest importance in tropical and developing areas of the world. However, the incidence of leptospirosis in temperate areas will likely increase in the coming years as a result of climate change leading to more frequent extreme weather events such as flooding and rising temperatures, and continued expansion of urban areas leading to increased contact with wildlife (Lau et al., 2010; Wasiński and Dutkiewicz, 2013). Leptospira are a complex family comprising 250 serovars grouped into 24 serogroups on the basis of their antigenic similarity (Adler and de la Peña Moctezuma, 2010). Leptospirosis infection occurs largely due to indirect transmission through contact with urine contamination of the environment (Levett, 2001). Survival of leptospires in the environment is influenced by factors such as temperature, precipitation, water quality, soil dampness and pH (Azócar-Aedo and Monti, 2016; Bierque et al., 2020; Lau et al., 2010). Additionally, leptospiral shedding in the environment is dependent on the presence of reservoir hosts such as rodents, dogs and livestock (Adler and de la Peña Moctezuma, 2010; Barragan et al., 2017), although maintenance host specificity to different serovars appears to vary and the mechanisms for this host/pathogen interaction are not clear (Adler and de la Peña Moctezuma, 2010).

Several associations between leptospirosis occurrence and agroecological factors have been proposed (Dhewantara et al., 2019; Lau et al., 2010; Mwachui et al., 2015a; Wasiński and Dutkiewicz, 2013) including increasing temperature (Zhao et al., 2016), areas with high rainfall (Sumi et al., 2017; Ward, 2002), outbreaks preceded by flooding (Barcellos and Sabroza, 2001; Gaynor et al., 2007; Dechet et al., 2012; Raghavan et al., 2012), and proximity to livestock (Mwachui et al., 2015a). Furthermore, a Brazilian study found relationships with environmental variables such as rainfall and temperature varied between serovars (Jara et al., 2019). However, there appears to have been no exploration of environmental factors at a serogroup or serovar level in temperate climates and therefore it is unclear if associations identified in previous research and focussed on tropical regions remain relevant in other areas of the world. Understanding the relationship between leptospira and the environment at a serovar or serogroup level has the potential to better inform control strategies, especially in areas where leptospirosis might become an emerging problem as a result of changing environmental conditions.

Currently, the important leptospira serogroups for dogs in Great Britain (GB) are not known, as the most recent serological survey took place 30 years ago (van den Broek et al., 1991). In this survey of dogs in Glasgow and Edinburgh, Canicola and Icterohaemorrhagiae serogroups were most frequently identified (van den Broek et al., 1991). For the past fifty years, control of canine leptospirosis in GB and continental Europe has centred around use of a bivalent vaccine containing Icterohaemorrhagiae and Canicola. More recently, in response to serological data from continental Europe, tetravalent vaccines were released, providing additional protection against two further serogroups: Australis and Grippotyphosa (Ellis, 2010; European Medicines Agency, 2017; Klaasen et al., 2013). Leptospirosis vaccine uptake appears to be high, with one study of dogs attending veterinary practices indicating that 81.5 % of dogs were vaccinated and of those vaccines administered, leptospirosis was the most frequent (Sánchez-Vizcaíno et al., 2018). However, it is currently unclear what the most important serogroups in GB are, where they are distributed and whether the composition of the tetravalent vaccine is representative of the serogroups prevalent in GB. Objectives of this study were therefore to (1) Determine serogroups of greatest important in GB (2) assess the spatio-temporal variation of leptospirosis test submissions and the three leptospirosis serogroups across GB, (3) explore associations between agro-environmental risk factors and distribution of different canine leptospirosis serogroups in GB, and (4) generate probability of presence maps for the different serogroups.

## 2 Materials and methods

### 2.1 Ethical approval

Ethical approval for the study was granted by the RVC Social Science Research Ethical Review Board (SR2019−0445).

### 2.2 Study area, population and disease data

The study area comprised GB and the study population consisted of all Microscopic Agglutination Test (MAT) and Polymerase Chain Reaction (PCR) test submissions for suspected leptospirosis in dogs to IDEXX laboratories between 1 January 2009 and 31 December 2018. PCR testing was performed on blood and/or urine and MAT was performed on serum. In accordance with European College of Veterinary Internal Medicine (ECVIM) guidelines (Schuller et al., 2015), a positive submission was defined as either a single MAT titre >1:800, paired titres with a four-fold increase in serology, or a PCR result reported as positive. The PCR and MAT results were recorded by IDEXX as either positive or negative but the MAT results additionally recorded antibody titres to each serovar represented in the panel. MAT and PCR test sensitivity and specificity is variable, however PCR generally exhibits higher sensitivity in early stages of the disease than the MAT due to the time required for seroconversion. One study using the same MAT cut-off threshold (>1:800) as our study on single samples reported 50 % sensitivity and 100 % specificity (Fraune et al., 2013). PCR test sensitivity has been reported at 91.6 % and specificity at 100 % in one study (Miotto et al., 2018), although performance varies depending on target gene and sample type (Schuller et al., 2015). Although MAT results are often reported as titre to serovars this has poor sensitivity due to cross-reactivity (44–46 %) whereas serogroup level reporting has much higher sensitivity (94 %) (Blanco et al., 2016; Levett, 2003; Public Health England, 2017). Therefore, dogs positive for the MAT test had the serogroup of the highest titre recorded as their primary infecting serogroup, rather than serovar.

In addition to test result, data on submitting clinic postcode and submission date were also extracted from the IDEXX database. Twenty submissions did not have postcodes and were therefore excluded from the spatial analysis but were retained for calculation of proportion of positive tests and exploration of the temporal distribution of disease. The remaining 1140 clinic postcodes were assigned cartesian coordinates and NUTS level 1 United Kingdom region codes (Office of National Statistics, 2017). As Northern Ireland had both the least cases (4, 1.3 %) and overall submissions (61, 1.6 %) these observations were removed from all further analyses leaving a total of 3759 observations. Submission date was categorised into seasons as follows: Spring (March, April, May), Summer (June, July, August), Autumn (September, October, November) and Winter (December, January, February).

### 2.3 Spatial analysis

#### 2.3.1 Visualisation of disease distribution

Point maps were created to show the spatial distribution of submissions, all leptospirosis cases, and individual serogroups. Kernel-smoothed maps of positive tests and all submissions were individually generated using the kernel density estimation function with a quartic kernel shape at a range of bandwidths (25−75 km), and the optimal bandwidth (30 km) chosen based on visual inspection. A kernel-smoothed ratio map showing density of cases adjusted for the underlying distribution of submissions was then obtained by dividing the kernel-smoothed surface of cases by that of submissions.

#### 2.3.2 Spatial and temporal cluster detection

Kulldorff’s spatial scan statistic was used to identify clusters of high or low risk for a positive diagnosis of leptospirosis amongst submissions, using SaTScan v9.6 (Kulldorff, 1997). Analysis was performed using three different models: the purely spatial Bernoulli model, purely spatial multinomial model and the seasonal scan statistic. In addition, to explore any potential differences in the location of clusters between the two types of test performed (PCR and MAT) the spatial scan statistic was applied to three different datasets: all submissions (n = 3986), only PCR submissions (n = 2270) and only MAT submissions (n = 1715). Furthermore, since different leptospira serogroups may have varied geographical distributions, the multinomial model (Jung et al., 2010) was used to differentiate clustering by serogroup. The seasonal scan statistic was used to identify temporal clusters of leptospirosis cases overall and for each serogroup individually, using month as the temporal unit and ignoring year of submission. Each analysis used a circular scanning window with maximal cluster size of less than 50 % of the population and Monte Carlo randomisation with 999 permutations (Kulldorff, 2015, 1997).

### 2.4 Sources and preparation of agroecological spatial data

All shapefiles and raster maps were generated and manipulated in QGIS v3.40 (Open Source Geospatial Foundation Project, 2020). Agroecological variables examined in this study were chosen on the basis of plausible biological mechanisms of transmission of the leptospira bacterium, previous research and the availability of open-source spatial datasets (Table 1), and included livestock density (cattle, sheep, pig, horse), rural-urban classification, land cover, temperature, rainfall, flooding and soil pH. Spatial layers were sourced for all variables (Table 1), converted to raster format if necessary before being clipped to the required extent using a GB shapefile (https://www.eea.europa.eu/data-and-maps/data/eea-reference-grids-2/gis-files/great-britain-shapefile), projected using the British National Grid (BNG) projection, and resampled to a resolution of 5 km^2^.

**Table 1:** Description and source of the agroecological variables included in the logistic regression and Maxent models of Leptospirosis distribution in Great Britain.

Variable	Description	Source
Cattle density	Density of cattle (heads/km^2^)	FAO Gridded Livestock of the World (2010) (FAO, 2018) (http://www.fao.org/livestock-systems/en/)
Sheep density	Density of sheep (heads/km^2^)	
Pig density	Density of pigs (heads/km^2^)	
Horse density	Density of horses (heads/km^2^)	
Dog density	Estimated density of dogs per postcode district	Animal and Plant Health Agency (Aegerter et al., 2017) https://data.gov.uk/dataset/ec8fc820−2e36−49d0-a09c-e2901e10b2e4/dog-population-per-postcode-district
Rural-urban classification	Rural-urban classification of Lower Super Output Areas (LSOA) of GB (6 classes)	Office of National Statistics (2012) (https://www.ons.gov.uk/methodology/geography/geographicalproducts/ruralurbanclassifications)
Land cover classification	Land cover of the UK (10 classes)	Centre for Ecology and Hydrology (2017) (https://www.ceh.ac.uk/services/land-cover-map-2015)
Temperature	Average annual temperature (^o^C) (2009−18)	Met Office HadUK-Grid^TM^ (Meterological Office et al., 2018; Hollis et al., 2019) (https://www.metoffice.gov.uk/research/climate/maps-and-data/data/haduk-grid/haduk-grid)
Rainfall	Average annual rainfall (mm) (2009−18)	
Flooding	Flood hazard map of maximum flood depth based on streamflow from past 10 years (m) (2006−16)	European and Global Flood Awareness Systems (EFAS and GloFAS) (Dottori et al., 2016) (https://www.globalfloods.eu)
Soil pH	Soil pH in water at 0 cm depth	International Soil Reference and Information Centre (2020)https://www.isric.org/explore/soil-geographic-databases

The livestock density, dog density, flood hazard, soil pH and landcover maps were used ‘as is’. The original rural-urban categories were mainly rural (1), largely rural (2), rural with significant urban (3), urban with city and town (4), urban with minor conturbation (5) and urban with major conturbation (6). These original categories were compressed further into predominantly rural (>80 % of population is classified as rural, classification codes 1 and 2), urban with significant rural (>50 - <80 % of the population is classified as urban, classification codes 3 and 4) and predominantly urban (>80 % urban population, classification codes 5 and 6) (DEFRA and Office of National Statistics, 2016; Eurostat, 2018).

For each year of the study (2009–2018), annual and monthly temperature and precipitation raster datasets were downloaded from the Met Office’s Had-UK gridded estimates (Dobson, 2020; Meterological Office et al., 2018). Rainfall and temperature data for each 5 km^2^ cell was averaged for each year over the study period to obtain average annual temperature and rainfall maps. Rainfall was also manipulated to compare monthly precipitation values against the standardised precipitation index (SPI). SPI measures how extreme precipitation is for a given location at a given time of year, relative to the historic conditions of that location (Mckee et al., 1993) thus providing a scale-independent comparison of the dryness or wetness over a specified time-period. To calculate SPI in this study, a gamma distribution was fitted to the lagged (accumulated precipitation over a 50-year historic period on any given month for any given location (i.e. the 5 km^2^ grid cells). The SPI of a given accumulation period was thus the inverse normal probability density of that amount of precipitation occurring. The dryness or wetness of a month was then categorised based on the SPI value into extreme wet (>2), severe wet (1.5 to −1.99), moderate wet (1.00 to −1.49), mild wet (0 to 0.99), mild drought (0 to -0.99), moderate drought (-1 to -1.49), severe drought (-1.50 to -1.99) and extreme drought (>-2) as defined by (McKee, et al., 1993). For this study the SPI categories were further grouped as severe & extreme wet, mild & moderate wet, mild & moderate drought and severe & extreme drought. The SPI was calculated for lag periods of 2 (SPI_Lag2), 3 (SPI_Lag3) and 12 months (SPI_Lag12) (Dobson, 2020). The Point Sampling Tool in QGIS v3.10 was used to extract the raster values of all variables to all positive and negative point locations (Open Source Geospatial Foundation Project, 2020).

### 2.5 Agroecological variable selection

Multicollinearity of all variables was assessed using the raw raster data for use in the ecological niche models by calculating the variance inflation factor (VIF) and correlation coefficients. A VIF of <10 and correlation coefficient of <0.7 was required for a variable to be retained for progression to the models (Pallant, 2010). Once variables were recategorized into quartiles, VIF and collinearity were then re-assessed with the same criteria, prior to inclusion in the regression model.

### 2.6 Approaches for exploring agroecological risk factors

Two separate modelling approaches were used to explore agroecological risk factors and leptospirosis. A multivariable logistic regression model was first used to explore agroecological and temporal risk factors associated with a positive test result amongst suspect cases. Due to the restricted and heterogenous sampling distribution of the laboratory submissions, ecological niche modelling (ENM) was then used to explore agroecological risk factors associated with leptospirosis presence, with a wider study background of the whole of Great Britain. Serogroup-specific agroecological preferences were then explored in MaxEnt as it is able to produce models of high predictive accuracy with small numbers of samples, which logistic regression would not be able to achieve (Wisz et al., 2008).

#### 2.6.1 Identification of agroecological risk factors

Multivariable binary logistic regression modelling was used to quantify the association between the spatial distribution of leptospirosis test submissions and the following agro-ecological variables: NUTS region, season, average annual temperature, average annual rainfall, SPI at 2-month, 3-month and 12-month lags, estimated dog density, livestock density (cattle, sheep, pig and horse), land-cover, urban-rural classification and soil pH. For the logistic regression model all variables were recategorized as follows: temperature, rainfall, dog density and soil pH were categorised based on quartiles while livestock density variables (cow, horse, sheep and pig) were categorised first as absent in an area corresponding to the submission (0-<1), and then submissions with livestock present were categorised based on terciles to create a total of four categories. Appropriate summary statistics were calculated for all variables included in the regression model. Due to the extent of some of the agroecological rasters, data was missing for some submissions: urban-rural classification (4.4 %,n = 165), soil pH (2.7 %, n = 100), cow, sheep and pig density (all 0.8 %,n = 30). These submissions were not retained for multivariable binary logistic regression.

Univariable logistic regression was used to quantify the association between each variable and a positive leptospirosis test result (either a MAT or PCR positive result). All variables with a p-value of <0.2 at the univariable level were taken forward to a multivariable logistic regression model. For the multivariable model, a manual backwards selection process was used whereby all variables carried forward from the univariable analyses were entered into the model and removed sequentially starting with the variable that was least significant. Likelihood ratio tests were used to compare models with and without the variables, and variables were retained if p < 0.05 from the likelihood ratio test (LRT). Confounding was assessed by comparing crude and adjusted odds ratios (ORs), and biologically plausible interactions between variables in the final model were assessed for significance and retained if the LRT result suggested they improved model fit (p < 0.05). Final model fit was assessed by the Hosmer-Lemeshow test, McFadden’s pseudo R^2^ and predictive ability assessed using area under the AUROC (Area Under the Receiver Operating Curve). Statistical significance was set at p < 0.05. Logistic regression was performed in R Studio v3.5.1 (R Core Team, Vienna, Austria) and model performance was evaluated with the pROC (Robin et al., 2021) and ResourceSelection (Lele et al., 2019) packages.

#### 2.6.2 Ecological niche modelling and predictive mapping

The maximum entropy algorithm, implemented in the MaxEnt software, was used to define the fundamental niches and estimate probability distributions of different leptospiral serogroups in GB (Phillips et al., 2017). MaxEnt uses location where a species has been recorded and the values of environmental variables at those locations to create a probability distribution of the environment in which a species has been found. This is compared with a probability distribution of the whole study area using randomly-generated background data points which characterise the environment of the full study area. Both probability distributions are built using the principles of maximum entropy (i.e. the most spatially different distribution that is possible). Ratios between these two probability distributions are calculated and used to build models quantifying the relative suitability of an area for the species (Elith et al., 2011). Multiple models are generated and the final model is chosen based on greatest similarity between the probability distribution of the species environment relative to the distribution of the environment of the study area (i.e. minimal relative entropy due to maximum entropy in their separate probability distributions) (Elith et al., 2011; Phillips et al., 2006).

As MaxEnt’s unit of analysis is the raster cell rather than individual point locations, all raster cells in the study area were classified as either positive (at least one positive submission) or negative (no positive submissions) resulting in 242 unique positives sites retained for analysis. In total, four distinct MaxEnt models were built: an AllCases_model and separate models for the Australis, Icterohaemorrhagiae and Sejroe serogroups. TheAllCases_model was built using the 242 unique geographic locations of positive MAT tests (titres ≥1:800) and PCR tests while the Australis (n = 23), Icterohaemorrhagiae (n = 24) and Sejroe models (n = 25) were built using positive MAT tests submissions which reported a single highest titre of ≥1:800. Many of the samples had titres >1:800 to multiple serogroups but since the relationship between highest titre and it being the infecting serogroup is unclear these were not retained. The following variables were included in the model: average annual temperature, average annual rainfall, livestock densities, soil pH, land cover classification, urban-rural classification and flooding. SPI and season were not included due to the temporal nature of these variables.

For each model, background points (n = 10,000) were randomly generated within the boundary of GB The model was run with ten replicates and 500 iterations at a convergence threshold of 0.00001, with each replicate using ten-fold cross-validation with random partitioning into training and test datasets, in order to obtain estimates of uncertainty around the fitted functions. MaxEnt’s built-in regularization method, which has been shown to be reliable and perform well (Hastie et al., 2009), was set to one to prevent over-fitting and lack of generalization (Phillips and Dudík, 2008). All feature classes of the algorithm were used to build the predictor variable response curves, and optimised models were generated using the jack-knife method which determines the predictive performance of the model with and without inclusion of a variable. If exclusion of the variable improved model predictive performance then the variable was removed from the final model. Performance of models built through the jack-knife method were evaluated through Aikake’s Information Criterion with small sample correction (AICc). The predictive performance of all final models was evaluated using threshold dependent assessment methods. Final model performance was assessed through AUC_TEST_ (maximum Area under Curve of Test data value), the True Skill Statistic (TSS) and Kappa Statistic. TSS and Kappa Statistic were calculated using the prevalence threshold.

## 3 Results

### 3.1 Test positive proportion and temporal distribution of leptospirosis

The test-positive proportion was 7.8 % (315/3986), with 9.4 % of PCR submissions positive (213/2270) compared with only 5.9 % of MAT submissions positive (102/1715). Of the MAT results with a high titre to a single serogroup (87.3 %, n = 89/102), the most frequently identified serogroups were Icterohaemorrhagiae (32.6 %, n = 30), Sejroe (28.3 %, n = 26) and Australis (27.2 %, n = 25).

**Fig. 1:**
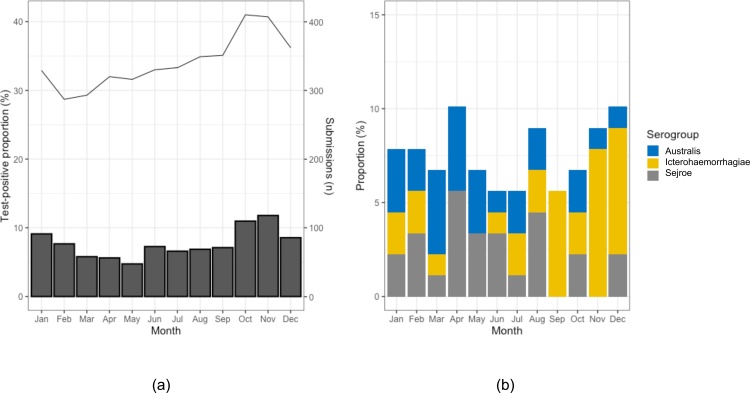
Leptospirosis MAT (Microscopic Agglutination Test) and PCR (Polymerase Chain Reaction) test submissions to IDEXX laboratories between 2009-18 showing (a) monthly test-positive proportion of leptospirosis (bars) and submissions (line), and (b) monthly proportion of individual serogroups (as identified by MAT testing as a proportion of MAT submissions that had only a single infecting serogroup identified).

The temporal distributions of positive tests and all submissions were similar (Fig. 1a), and the test-positive proportion was highest in the Autumn months of November (11.8 %, 48/407) and October (11.0 %, 45/410); more than double that of the Spring months of April (5.6 %, 18/320) and May (4.7 %, 15/316; Fig. 1a). Moreover, the seasonal scan statistic identified a significant temporal cluster of increased relative risk of (RR) a positive result from October to January, during which a submission was 1.5 times more likely to have a positive result than the rest of the year (RR = 1.54, p = 0.004). Serogroup proportions appeared to differ seasonally between individual serogroups with Australis and Sejroe comprising a higher proportion of positive MAT results in the first half of the year, while Icterohaemorrhagiae comprised a higher proportion of positive MAT results in the latter half of the year, with the proportion of positive MAT tests 4 times higher in September, November and December than the rest of the year (Fig. 1b). Moreover, the seasonal scan statistic identified a significantly increased risk of Icterohaemorrhagiae serogroup being associated with positive tests from September to December (RR = 3.58, p = 0.01). No significant temporal clusters were identified for the other two serogroups.

### 3.2 Spatial distribution of leptospirosis

**Fig. 2:**
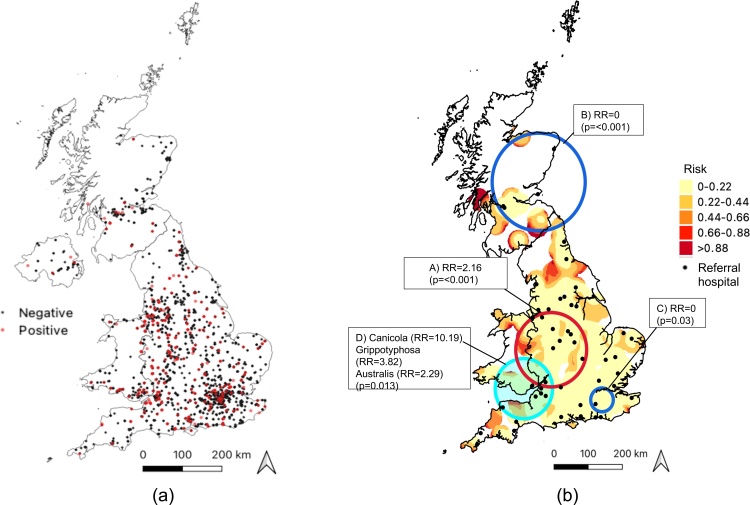
(a) Point map showing location of positive (red dots) and negative submissions (black dots). (b) Kernel density ratio surface displaying the kernel-smoothed density of leptospirosis cases after adjusting for the distribution of all submissions (bandwidth 30 km; resolution 5km^2^) overlaid with the location of four significant spatial clusters of leptospirosis cases: a single high-risk cluster (A; p < 0.001) and two low-risk clusters (B; p < 0.001 and C; p = 0.03). Cluster D) indicates significantly higher proportions of Canicola, Grippotyphosa and Australis serogroup cases (p = 0.013). Black dots indicate location of referral veterinary hospitals.

Although the South East (17.4 %, n = 693) and South West England (14.1 %, n = 563) reported the highest proportion of submissions, the test-positive proportion was highest in the West (12.5 %, n = 43/344) and East Midlands (11.5 %, n = 26/226) (Table 1). Distribution of submissions was spatially heterogeneous being largely concentrated in south and central England with patchy submissions in northern England, Scotland and Wales (Fig. 2a). However, after adjusting for the distribution of submissions, the kernel-smoothed ratio map showed areas with the highest ratio of positive tests to submissions included areas in western Scotland, the Wales/England border and northern and south-western England (Fig. 2b). Moreover, these high-density areas seldom coincided with large numbers of referral hospitals (Fig. 2b). Three significant spatial clusters of positive tests amongst PCR submissions were identified – one high- and two low-risk - but no clusters among MAT submissions. The high-risk cluster was centred in the county of Shropshire and submissions from within this cluster were roughly twice as likely to be positive for leptospirosis than submissions outside the cluster (RR = 2.16, p = 0.005, Fig. 2b, red circle A). The two low-risk for positive tests clusters were centred on north-eastern Scotland and South East England where submissions from these areas included no cases (RR = 0, p = 0.01 and 0.03 respectively, Fig. 2b, blue circles B and C).

**Fig. 3:**
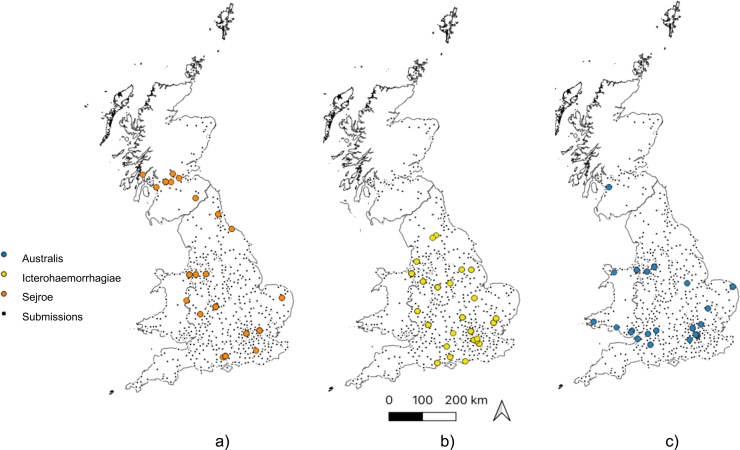
Point maps showing distribution of all Leptospirosis-positive submissions (grey dots) in Great Britain (2009-2018) together with the distribution of the three most common serogroups (coloured circles): (a) Sejroe (n = 26), (b) Icterohaemorrhagiae (n = 30) and c) Australis (n = 25).

The Sejroe serogroup predominated in Scotland and northern England (Fig. 3a), while both the Icterohaemorrhagiae (Fig. 3b) and Australis (Fig. 3c) serogroups were most commonly seen in central England. Australis was additionally the main serogroup identified in Wales. Furthermore, the multinomial spatial scan statistic identified one significant cluster of serogroups (p = 0.013) centred over southern Wales and the Devon-Somerset area (Fig. 2b, circle D in cyan). Compared to submissions outside this cluster, positive submissions from within the cluster were ten times more likely to be Canicola (RR = 10.19), almost four times as likely to be Grippotyphosa (RR = 3.82) and just over twice as likely to be Australis (RR = 2.29).

### 3.3 Agroecological risk factors for leptospirosis

Cow and pig density exhibited collinearity (correlation = 0.8); all other variables were not correlated (correlation = 0−0.4). All 14 were included in the univariable analyses (Table 2). Six variables were taken forward to the multivariable model: season (p = 0.002), region (p = 0.003), urban-rural classification (p = 0.06), average annual temperature (p = 0.12), dog density (p = 0.12) and SPI_Lag12 (p = 0.12) although only three of these remained in the final multivariable model: season (p < 0.001), region (p = 0.004) and urban-rural classification (p = 0.04) (Table 3). The final model showed that a positive leptospirosis test result was nearly twice as likely to occur in Autumn (OR = 1.86, p < 0.001) and one and a half times as likely to occur in Winter (OR = 1.51, p = 0.03), than in Spring. Compared to South East England, submissions were more than twice as likely to be positive in the West (OR = 2.32, p < 0.001) and East Midlands (OR = 2.20, p = 0.002). When compared to urban areas, submissions from areas with an intermediate urban-rural area had increased odds of a positive test result (OR = 1.52, p = 0.02). There were no significant interactions in the model and the final model showed acceptable goodness-of-fit (Hosmer-Lemeshow chi-square test = 7.97, p = 0.44) and moderate predictive ability (AUROC 0.64, 95 % CI 0.61−0.70, McFadden’s Psuedo-R^2^ = 0.02).

**Table 2:** Descriptive statistics of leptospirosis positive and negative submissions and environmental variables examined in univariable analysis. Leptospirosis positive dogs (n = 290) had a positive MAT or PCR test result from IDEXX Laboratories 2009-18. Negative dogs (n = 3469) had a negative PCR or MAT test result from IDEXX Laboratories submissions between 2009-18. Variables are listed from most to least significant.

Variable	Category	Submissions (n = 3986)		OR (95 %CI)	P-value	
		Positive ((%) n)	Negative ((%) n)		Wald	LRT
Season	Spring	15.9 (46)	23.6 (819)	1		0.002
	Summer	23.1 (67)	24.9 (864)	1.38 (0.94−2.03)	0.10	
	Autumn	36.2 (105)	27.8 (965)	1.94 (1.35−2.77)	<0.001	
	Winter	24.8 (72)	23.7 (821)	1.56 (1.07−2.29)	0.02	
NUTS Region	South East England	14.5 (42)	17.8 (618)	1		0.02
	North East England	2.1 (6)	2.7 (94)	0.95 (0.39−2.30)	0.89	
	North West England	12.8 (37)	12.7 (441)	1.23 (0.78−1.95)	0.37	
	Yorkshire & Humber	5.2 (15)	5.6 (194)	1.14 (0.62−2.09)	0.67	
	East Midlands	9.0 (26)	5.3 (185)	2.07 (1.23−3.44)	0.006	
	West Midlands	13.4 (39)	7.8 (272)	2.11 (1.33−3.46)	0.001	
	East of England	11.0 (32)	13.0 (451)	1.10 (0.69−1.75)	0.69	
	London	4.1 (12)	5.9 (208)	0.90 (0.47−1.74)	0.76	
	South West England	14.5 (42)	14.3 (495)	1.25 (0.80−1.95)	0.33	
	Wales	8.6 (25)	8.1 (280)	1.31 (0.79−2.20)	0.30	
	Scotland	4.8 (14)	6.7 (231)	0.89 (0.45−1.56)	0.72	
Urban-rural	Predominantly urban	63.4 (184)	69.0(2394)	1		0.06
	Urban with significant rural	18.3 (53)	13.8 (480)	1.44 (1.04−1.98)	0.03	
	Predominantly rural	14.8 (43)	12.7 (440)	1.27 (0.90−1.80)	0.18	
Average annual temperature (^o^C)	0−9.99	24.5 (71)	23.4 (812)	1		0.12
	9.99−10.4	31.7 (92)	26.8 (930)	1.13 (0.82−1.56)	0.46	
	10.4−10.7	25.2 (73)	25.9 (900)	0.93 (0.66−1.30)	0.67	
	10.7−11.53	18.6 (54)	23.8 (827)	0.75 (0.52−1.08)	0.12	
SPI_Lag12	Mild & moderate wet	42.8 (124)	40.2 (1394)	1		0.12
	Severe & extreme wet	16.6 (48)	15.2 (527)	1.02 (0.72−1.45)	0.89	
	Mild & moderate drought	36.6 (106)	42.3 (1467)	0.81 (0.62−1.06)	0.13	
	Severe & extreme drought	4.1 (12)	2.3 (81)	1.67 (0.88−3.14)	0.11	
Estimated dog density (dogs/km^2^)	0- 413	23.8 (69)	25.1 (871)	1		0.12
	413- 692	20.0 (58)	25.3 (878)	0.83 (0.58−1.20)	0.33	
	692- 1143	27.0 (79)	24.8 (859)	1.16 (0.83−1.63)	0.39	
	1143- 2700	29.0 (84)	24.8 (861)	1.23 (0.88−1.72)	0.22	
Average annual rainfall (mm)	0- 562.8	26.6 (77)	27.9(969)	1		0.34
	562.8- 658.3	26.2 (76)	22.5 (780)	1.23 (0.88−1.71)	0.23	
	658.3- 745.4	20.7 (60)	23.9 (828)	0.91 (0.64−1.29)	0.61	
	745.4- 2174	26.6 (77)	25.7 (892)	1.09 (0.78−1.51)	0.62	
Horse density (heads/km^2^)	Absent	27.9 (81)	24.2 (841)	1		0.27
	1−2	29.0 (84)	24.7 (858)	1.02 (0.74−1.40)	0.92	
	2−4	23.4 (68)	25.5 (885)	0.80 (0.57−1.12)	0.19	
	4−16	19.7 (57)	25.0 (868)	0.68 (0.48−0.97)	0.03	
Soil pH	4.6−5.7	26.6 (77)	26.8 (930)	1		0.36
	5.7−6.0	24.8 (72)	23.4 (812)	1.20 (0.88−1.65)	0.26	
	6.0−6.4	24.8 (72)	27.6 (958)	0.89 (0.64−1.23)	0.48	
	6.4−7.4	20.0 (58)	19.6 (680)	1.07(0.76−1.51)	0.69	
Cow density (heads/km^2^)	Absent	41.7 (121)	42.4 (1471)	1		0.46
	1−54	6.9 (20)	7.3 (253)	0.96 (0.59−1.57)	0.87	
	54−313	22.1 (64)	25.0 (868)	0.90 (0.65−1.23)	0.49	
	313−555	28.3 (82)	24.5 (850)	1.17 (0.88−1.57)	0.29	
SPI_Lag3	Mild & moderate wet	45.5 (132)	46.6 (1617)	1		0.53
	Severe & extreme wet	9.0 (26)	6.8 (235)	1.36 (0.87−2.11)	0.18	
	Mild & moderate drought	40.0 (116)	41.4 (1436)	0.99 (0.76−1.28)	0.94	
	Severe & extreme drought	5.5 (16)	5.2 (181)	1.08 (0.63−1.86)	0.77	
Land cover	Broadleaf woodland	0.0 (0)	0.7 (23)	0 (0-infinity)	0.97	0.57
	Built up areas, gardens	68.6 (199)	68.7(2383)	1		
	Arable	6.9 (20)	7.6 (262)	0.91 90.57−1.47)	0.71	
	Improved grassland	24.5 (71)	22.8 (791)	1.07 (0.81−1.42)	0.62	
	Coastal	0.0 (0)	0.3 (9)	0(0-inf)	0.99	
SPI _Lag2	Mild & moderate wet	48.6 (141)	48.7 (1688)	1		0.70
	Severe & extreme wet	7.2 (21)	5.6 (195)	1.29 (0.80−2.09)	0.30	
	Mild & moderate drought	39.0 (113)	39.9 (1383)	0.98 (0.76−1.27)	0.87	
	Severe & extreme drought	5.2 (15)	5.9 (203)	0.88 (0.51−1.54)	0.66	
Pig density (heads/km^2^)	Absent	41.7 (121)	42.3 (1469)	1		0.80
	1−62	7.2 (21)	7.4 (255)	0.80 (0.55−1.16)	0.23	
	62−304	23.1 (67)	25.0 (867)	1.25 (0.90−1.75)	0.19	
	304−557	26.9 (78)	24.5 (851)	1.13 (0.81−1.59)	0.44	
Sheep density (heads/km^2^)	Absent	41.7 (121)	42.3 (1468)	1		0.86
	1−61	6.6 (19)	7.5 (259)	0.89 (0.54−1.47)	0.75	
	61−305	24.1 (70)	24.8 (860)	0.99 (0.73−1.34)	0.94	
	305−556	26.6 (77)	24.6 (855)	1.09 (0.81−1.47)	0.56	

SPI= standardised precipitation index.

**Table 3:** Final multivariable logistic regression model of variables associated with a positive diagnosis of leptospirosis amongst laboratory submissions between 2009 and 2018. Only dogs with complete records were retained for the multivariable model (n = 3594).

Variable	Category	OR (95 %CI)	P-value
Season	Spring	1	
	Summer	1.32 (0.89−1.96)	0.17
	Autumn	1.86 (1.29−2.69)	<0.001
	Winter	1.51 (1.02−2.23)	0.04
NUTS region	South East England	1	
	North East England	1.09 (0.45−2.67)	0.85
	North West England	1.22 (0.76−1.94)	0.41
	Yorkshire and Humber	1.26 (0.68−2.35)	0.46
	East Midlands	2.20 (1.31−3.71)	0.003
	West Midlands	2.32 (1.45−3.71)	<0.001
	East of England	1.21 (0.75−2.00)	0.41
	London	0.99 (0.50−1.94)	0.97
	South West England	1.18 (0.75−1.89)	0.49
	Wales	1.34 (0.802.25)	0.27
	Scotland	1.09 (0.56−2.12)	0.81
Urban-rural classification	Predominantly urban	1	
	Urban with significant rural	1.52 (1.08−2.14)	0.02
	Predominantly rural	1.31 (0.92−1.93)	0.13

### 3.4 Ecological niche models of leptospirosis

All four models had AUROC values in the good to excellent range (0.80−0.86). The Australis model had the best predictive ability, with the highest AUROC (0.86), high sensitivity and specificity (0.85 and 0.63), while the Sejroe model had the lowest AUROC (0.80) and TSS value (TSS = 0.44). The Icterrohaemorrhagiae model had the highest TSS value (0.53). Sensitivity was higher than specificity in all models suggesting all models were all better at identifying where disease occurred rather than where it did not occur. All models had a moderate TSS (>0.4) (Landis and Koch’s (1977) (Table 4).

**Table 4:** Performance parameters of four Maxent models of canine leptospirosis distribution in Great Britain. Models were based on the distribution of all positive samples (AllCases), and the distribution of the Australis, Icterohaemorrhagiae and Sejroe serogroups.

Model performance index	AllCases	Australis	Icterohaemorrhagiae	Sejroe
**Area Under Curve Reciever Operator Curve (AUROC)**	0.81	0.86	0.84	0.80
**Threshold value (prevalence)**	0.24	0.15	0.16	0.28
**Kappa statistic max**	0.27	0.29	0.25	0.27
**Sensitivity**	0.89	0.85	0.90	0.86
**Specificity**	0.50	0.63	0.63	0.58
**True Skill Statistic (TSS)**	0.41	0.48	0.53	0.44

Variable retention and importance varied between the models although average annual temperature was the most important variable across all four models (Table 5) and accounted for almost half (47.8 %) the variation in distribution of all cases (AllCases_model). However, its contribution varied widely between the serogroup models from accounting for 58.5 % of the variation in distribution of Sejroe cases to only 35.9 % in the Icterohaemorrhagiae model. The second most important variable was different for each of the models: landcover (29.1 % AllCases_model and 29.0 % Icterohaemorrhagiae), horse density (22.8 % Australis) and soil pH (24.8 %, Sejroe). The AllCases_model and Australis models retained the most variables (5) of all the models, whereas the Sejroe model kept the least (3). Land cover, average annual rainfall and dog density were retained in three of the models, while pig density and soil pH were only retained in one model each (Icterohaemorrhagiae and Sejroe models, respectively). None of the final models retained flooding or sheep density.

**Table 5:** Percentage contribution of the agroecological variables retained in the AllCases_model and three individual serogroup Maxent models of leptospirosis in GB built Shaded cells indicate variables not retained in the final model.

**Fig. 4:**
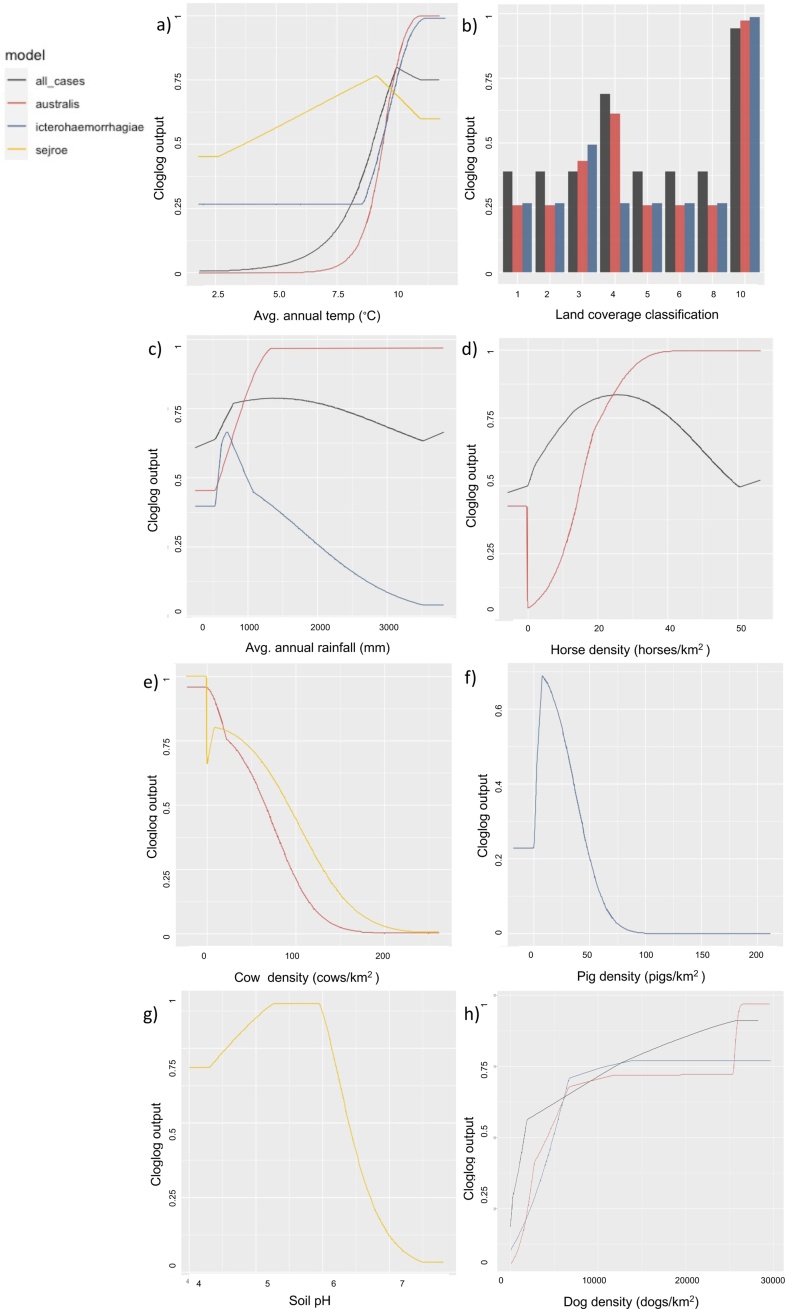
Response curves describing probability of presence of of canine leptospires over the range of values of each predictor variable in four maximum entropy (MaxEnt) models (i.e., values of other variables kept constant values) for the environmental variables retained in each of the final models. The x-axis displays the range of the agroecological variable, and the y-axis indicates probability of presence (scale: 0-1). (a) Annual average temperature, (b) landcover, (c) annual average rainfall, (d) horse density, (e) cow density, (f) pig density, (g) soil pH and (h) dog density. Colours shown in top left-hand corner indicate the model: AllCases_model = black, Australis model = red, Icterohaemorrhagiae model = blue and Sejroe = yellow. Land cover classifications are 1=broadleaf woodland, 2=coniferous woodland, 3=arable, 4=improved grassland, 5=seminatural grassland, 6=mountain heath, 8=coastal and 10=built up areas and gardens.

Fig. 4 shows the response curves for the different agroecological variables retained in each of the final models. In all models, as average annual temperature increased, probability of presence increased up to a maximum probability at 11 °C, although the average annual temperature with highest probability of presence varied amongst serogroup models (Fig. 4a). The Sejroe model had the lowest average annual temperature peak of probability (9 °C) whereas the Australis and Icterohaemorrhagiae models both had a similar and warmer average annual temperature peak (11 °C: Fig. 4a). For the 3 models that retained land coverage (AllCases, Australis and Icterohaemorrhagiae) built up areas/gardens was the most important class (Fig. 4b). Improved grassland also had high probability of presence for the AllCases_model. The association between rainfall and the three models that retained it as a variable differed (Fig. 4c). In the Icterohaemorrhagiae model probability increased up to a peak of annual rainfall at 750 mm and decreased sharply beyond that (Fig. 4c). For the Australis model higher annual rainfall was associated with a high probability of presence whereas for the AllCases_model the relationship with rain appeared to be a combination of these two models with a sharp rise in probability at 750 mm per year and then gradually decreasing at higher levels of rainfall (Fig. 4c).

In the AllCases_model as horse density increased up to about 20 heads/km^2^ probability rose but then decreased at higher densities (Fig. 4d). However, in the Australis model, as horse density increased, probability of Australis presence increased and remained high (Fig. 4d). In both the Australis and Sejroe models these serogroups were absent in areas of high cattle density, beyond their initial high probability between 0–50 cows/km^2^, although cattle density did not contribute substantially to the Australis model (Fig. 4e). Low pig density was associated with a moderate probability of Icterohaemorrhagiae presence (∼25 pigs/km^2^) (Fig. 4f) but at higher pig densities (>100 heads/km^2^) Icterohaemorrhagiae was not present. Finally, as acidity of soil pH decreased towards an optimal pH of 5.5–6.0, probability of Sejroe presence increased, but as soil pH approached a neutral pH of 7 probability of presence dropped (Fig. 4g). The AllCases_model, Australis and Icterohaemorrhagaie models all had increased probability of presence as density of dogs per kilometre increased. For the Australis model, highest probability was seen at the highest dog density (3000 dogs/km^2^) whereas for the other models highest probability of presence was at approximately 2500 dogs/km^2^ (AllCases_model) and 1200 dogs/km^2^ (Icterohaemorrhagiae model).

**Fig. 5:**
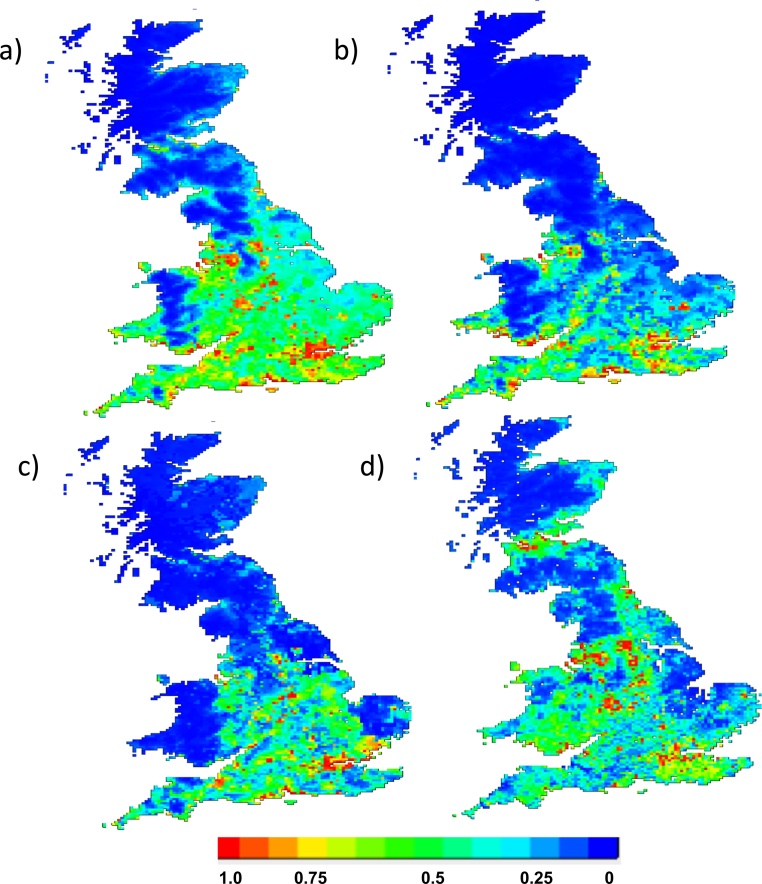
Maps illustrating probability of presence of canine leptospirosis in Great Britain, for all cases (a) and individual serogroups (b-d) as determined by a maximum entropy (MaxEnt) model with cross-validation and averaged across ten model replicates. a)AllCases_model, b) Australis serogroup, c) Icterohaemorrhagiae serogroup and d) Sejroe serogroup.

Although predicted distribution varied between the different serogroups each model followed a general pattern of lower probability (blue/green colours) in Scotland and northern and eastern England, and higher probability in the south-east of England (red/orange) (Fig. 5). TheAllCases_model (Fig. 5a) and Australis model (Fig. 5b) additionally found large areas in Kent/Sussex areas to be of high probability also. The Australis probability map (Fig. 5b) suggested that Australis cases appeared to be associated with coastal areas, or cities with rivers, more so than other serogroups. This can be seen in the areas of high probability for Australis present along coastal southern Wales and England. Most of Wales appeared unsuitable for all serogroups, with the exception of Australis in coastal southern Wales (Fig. 5b). From the map generated by the Icterohaemorrhagiae model (Fig. 5c), it is apparent that all of Wales had a low probability of Icterohaemorrhagiae presence. In the Icterohaemorrhagiae (Fig. 5c) and Sejroe maps (Fig. 5d) there were isolated areas of high probability of presence in the Midlands and lower northern regions of England. The probability map for the presence of Sejroe serogroup (Fig. 5d) appeared most patchy of all, with small areas of high probability in south-eastern, central and north-eastern England and lowland Scotland. However, the Sejroe serogroup was the only one to have a high probability of presence in Scotland.

## 4 Discussion

Understanding the spatial distribution of leptospirosis at the serogroup level together with environmental factors associated with disease is important, due to direct dog-dog transmission being rare and environment-acquired infections being of greater significance (Picardeau, 2013). This study explored variation in the spatial and seasonal patterns, and the agroecological risk factors of canine leptospirosis for laboratory submissions in GB, particularly focussing on the serogroup level. There was a significantly increased risk of a diagnosis of leptospirosis in Autumn and Winter, and around the England-Wales border; although the highest predicted probability of presence for leptospirosis was identified in southern to central England. The Australis, Icterohaemorrhagiae and Sejroe serogroups displayed different spatial and temporal distributions, and the agroecological factors associated with the presence of canine leptospirosis and the relationship with these factors also varied between serogroups, suggesting that different serogroups have different ecological preferences. Possibly of greatest importance though is that the serogroup with the second highest prevalence (Sejroe, 28.3 %, n = 26) is not included in the tetravalent vaccines currently used by GB veterinarians.

### 4.1 Spatial and temporal distribution

In this study, the number of submissions and the proportion of positive tests varied between months and regions. This spatio-temporal variation could be attributed to agroecological risk factors and/or increasing awareness of the disease by veterinarians and/or owners, potentially due to recent outbreaks. Increased awareness of the disease could manifest as veterinarians undertaking leptospirosis testing more widely in dogs with a varying index of suspicion. More widespread use of testing could then lead to a decrease in the test-positive proportion for a region. Potentially this pattern is seen in South West England, where an outbreak of canine leptospirosis was reported in Somerset in 2014 (Wilson et al., 2015). In this region the number of leptospirosis submissions was high, yet the test-positive proportion was lower than other regions in this study.

The test-positive proportion, number of submissions and risk of a positive result were increased in this study in Autumn. Despite the number of submissions being higher in Autumn the test-positive proportion increased and these findings suggest that canine leptospirosis might exhibit seasonal variation, with increased disease in Autumn; a pattern identified in previous studies (Alton et al., 2009; Hennebelle et al., 2010; Lee et al., 2014; Major et al., 2014; Raghavan et al., 2011; Smith et al., 2019; Ward, 2002).

The test-positive proportion varied regionally in this dataset ranging from 5.2 (London) to 12 % (East and West Midlands) and this heterogenous spatial distribution was supported by the locations of a high-risk cluster for positive tests centred on Shropshire and two low-risk clusters identified in South East England and eastern Scotland (Fig. 2). Diagnostic tests may be more likely to be submitted by referral hospitals than primary care clinics, and as there are more referral centres located in the high-risk clusters than the low-risk clusters, this could suggest that these clusters may simply be an artefact of the distribution of such hospitals. However, the fact that the areas of high density of positive test rate shown on the kernel-smoothed ratio surface seldom coincide with areas where there are more referral hospitals suggests that the clustering of positive tests observed in this study was unlikely to be the result of clustering around referral centres. Serogroup distribution varied spatially, with the Sejroe serogroup being the predominant serogroup in Scotland and northern England (Fig. 2 circle D and Fig. 3). However, this dataset represents laboratory submissions only rather than a random sample across the dog population of GB, spatial and seasonal distribution should be interpreted with this in mind.

### 4.2 Risk factors and ecological niches

This study used two different modelling algorithms to explore the relationship between a range of agro-ecological variables and disease distribution. Although the regression model described in Section 3.3 was used to identify risk factors for a positive test result there were limitations associated with this approach. Firstly, as the submissions dataset used for the regression model was heterogeneously distributed with a focus on south and central England, the risk factors for a positive test identified in the regression model would have likely been biased towards these areas, despite positive submissions being identified in Wales and Scotland. Additionally, robust regression models could not be generated for individual serogroups owing to the comparatively few samples with this information (<30 for each serogroup). Therefore, in addition to the regression model, ecological niche models were developed using the maximum entropy algorithm, as implemented in the Maxent software. The benefits of this combined approach were three-fold. Firstly, the logistic regression model allowed for the exploration of variables with a temporal nature (season and SPI) – something which could not be achieved in the Maxent model. Secondly, the Maxent model allowed for the exploration of risk factors associated with the distribution of specific serogroups as, unlike regression models, MaxEnt has been shown to have good predictive accuracy even with small samples; as few as ten in some studies (Wisz et al., 2008). And lastly, distribution of submissions was spatially heterogeneous being largely concentrated in south and central England with a lack of submissions in Scotland, Wales and northern England despite cases being observed in these regions. Using this biased distribution of negative submission as the comparison could potentially lead to truncated or incorrect modelling of the ecological niche. Instead, modelling the agroecological associations of leptospirosis and individual serogroups with randomly generated background data points that cover the entire study area homogenously as the comparison rather than heterogeneously distributed negative submissions ensures that agroecological conditions in under-represented areas are included in the analysis. However, although Maxent allows for inclusion of under-represented areas in the background study area it cannot compensate for the heterogenous and biased distribution of positive submissions and this caveat should be borne in mind when interpreting the models. Although background data could have be generated with use of a bias grid from distribution of submissions this would not have allowed us to determine if there are additional areas in GB that are suitable for leptospirosis but not reported yet.

The regression model identified region, season and urban-rural classification as being significantly associated with a positive test result, while the Maxent models suggest that, in general, leptospira transmission favoured regions of GB with a warmer average annual temperature (south and central England) and urban/suburban land types, with serogroup-specific relationships to livestock densities and soil pH. Risk factors identified in the logistic regression of submissions represent risk factors associated with a positive test result amongst suspect cases. This differs from the outputs of ENM which identify areas across GB with similar agroecological conditions to areas where cases have been located. Associations with agroecological variables should be interpreted in this light.

The lack of agreement between variable retention between these two different aspects of the study is likely multifactorial. Firstly, temporal variables (season and SPI) were not suitable for inclusion in the ENM because MaxEnt uses spatial data only, in the form of a gridded dataset that uses either information averaged across the whole study period (eg. Average annual temperature) or from a single time point (eg. Livestock density). Therefore, variables used in logistic regression models that are associated with the date of submission cannot be used in MaxEnt. Additionally, season and region are both very broad variables that encompass information from multiple agroecological variables (e.g., within a region livestock densities and climatic conditions will vary) and the regression model will likely have identified broad variation while the Maxent model may have been able to differentiate between finer variation. The seasonal component to the disease, suggested through spatial scanning statistic results, is not accounted for in the MaxEnt models and this may be why temperature is the most important variable in MaxEnt. Temperature may partially encapsulate some of the seasonal effect. A logistic regression model built with only the variables available for the MaxEnt model was attempted, to facilitate more direct comparisons, but variables were not significant at the multivariable level.

Comparison between the two models is limited largely because the background points generated by MaxEnt span the whole of GB whereas logistic regression used the more restrictive or artificial distribution of laboratory submissions which, in this instance were biased towards South and central England. Furthermore, all variables in the regression model were included as categorical variables while only two of the variables in the MaxEnt model were categorical. The use of continuous variables in the MaxEnt model likely allowed the algorithm to identify fine-scale differences that were lost when variables were categorised and allowed a more detailed picture of the agroecological associations to emerge.

All leptospira ENMs identified increased probability of presence as average annual temperature increased, the peak was highest for Australis and Icterohaemorrhagiae serogroups (11 °C) and lowest for the Sejroe serogroup (9 °C). The temperature range preference for leptospira identified here suggests that the ecological niche of the Australis and Icterohaemorrhagiae serogroups is mostly restricted to the southern half of GB (Fig. 5). However, the slightly lower temperature and acidic soil pH ecological preferences reported in the Sejroe model suggest that northern England and Scotland are suitable for the Sejroe serogroup as indicated by the fact that Sejroe was the predominant serogroup seen in northern England and Scotland in this study (Fig. 3).Warmer temperatures have been associated with leptospirosis in previous MaxEnt studies (Zhao et al., 2016) and preferential temperature ranges have reportedly varied between serovars (Jara et al., 2019). A preference for warmer annual temperatures likely reflects increased leptospire survival time in warmer weather but additionally higher temperatures can lead to increased water-based activities, thereby facilitating increased transmission (Lau et al., 2010). Climate projections for the UK indicate rising temperatures and also increased extreme weather events such as heavy rainfall, flooding and droughts. The annual average temperature for 2019 for all countries in the UK was 0.5 °C warmer versus the 1981−2010 average (Kendon et al., 2020). The lower bound estimate on average annual temperature increase is 0.5 °C rise by 2050 (Met Office, 2019). Although these predictions from these ENMs have not been explored versus different climate scenarios, based on our finding of higher probability for leptospirosis presence in areas with warmer temperatures, increasing temperatures may result in a more widespread distribution of leptospirosis in GB and variation in serogroup distribution.

Increased probability in urban/suburban areas (identified in both MaxEnt and binary logistic regression models) could reflect increased contact between dogs and rodents or other reservoir hosts and increased usage of same shared walking spaces (Lau et al., 2010; Raghavan et al., 2011). Meta-analysis by Mwachui et al. (2015a), 2015b identified conflicted study findings over urban-rural leptospirosis risk. In regression analyses increased odds of a positive test result amongst suspect cases in urban with significant rural areas was identified, and when agroecological risk factors across GB were explored in MaxEnt urban/suburban areas had increased risk. Since most submitting veterinary practices were in urban/semi-urban areas (∼75 % submissions) this will likely have impacted these results. Therefore, due to the underlying biases of submissions from urban/semi-urban areas we consider the inclusion of this variable to be for the purpose of hypothesis generation rather than confirmation of urban/rural associations.

This study showed that individual serogroups were associated with distinct livestock species. The Australis model found increased horse density to be associated with higher probability. L. *Bratislava*, part of the Australis serogroup, is the serovar most frequently identified in horses and is host-adapted with high rates of asymptomatic carriage reported previously (Houwers et al., 2011; Verma et al., 2013). As it is a clinically important serovar for dogs, identified in an outbreak in dogs in Somerset (Wilson et al., 2015), the role of horses in transmission of leptospirosis in GB merits further exploration. Additionally, low densities of pigs (up to 100 heads/km^2^) in the Icterohaemorrhagiae model was associated with high probability of presence. Lower densities of pigs may reflect areas with small holdings or smaller commercial farms predominating. Biosecurity and vaccination may be less commonplace in smaller scale production and therefore increased environmental shedding of leptospires may occur. Indeed, a recent outbreak on a pig farm in England was attributed to L. *Icterohaemorrhagiae* (Animal Plant Health Agency, 2018). Areas with high dog densities were associated high probability of presence in the AllCases_model, Icterohaemorrhagiae and Australis models. This could reflect increased indirect transmission opportunity by a higher density of dogs frequenting shared spaces such as parks and popular walking routes.

Knowledge of serogroup-specific niches may improve understanding of local disease transmission and implementation of control strategies. The composition of the existing tetravalent vaccines provide coverage for only two of the three most prevalent serogroups identified here (Icterohaemorrhagiae and Australis) and omits the Sejroe serogroup which seems to have the widest range in GB. For areas with high probability of Sejroe transmission there is no existing leptospirosis vaccine that offers protective immunity, as cross-serogroup protection is rarely conferred (Klaasen and Adler, 2015). The variability between serogroup niches highlights the importance of ongoing disease surveillance, ensuring that the coverage provided through vaccination is in alignment with locally relevant field strains. One such example of this is inclusion of L. *Pomona* in the US version of the leptospirosis tetravalent vaccine, due to its importance in the US (Ghneim et al., 2007), replacing the Australis serogroup which is included in the European version of the vaccine due to its prevalence there (Ellis, 2010; Francey et al., 2020; Renaud et al., 2013).

### 4.3 Limitations

Use of retrospective laboratory submission datasets have several key limitations. There are likely to be several biases underlying the dogs chosen for testing. These dogs are more likely to be either severely affected by the disease or have a more classical leptospirosis presentation of hepatic or renal dysfunction. Dogs with milder disease or more obscure presentations may not be considered for diagnostic testing, yet dogs with less severe or absent clinical signs are likely to be significant contributors to the environmental transmission of the disease and would present a zoonotic risk to pet owners if they are not being treated appropriately. Indeed, surveys of healthy dogs identified 1.5–7 % were shedding leptospires in their urine (Llewellyn et al., 2016; Rojas et al., 2010). Additionally, diagnostic tests are expensive, so higher-income clients or insured pets are more likely to be tested (O’Neill et al., 2014). This means that areas found to be of increased or reduced risk may be partially attributable to socio-economic factors, rather than only true disease/environmental reasons. The effect of socio-economic status is possibly reflected in this work with more frequent testing, higher test-positive proportions and increased probability of presence of leptospirosis seen in less deprived, more affluent southern and central England versus further north in England (Department for Communities and Local Government, 2015; Legatum Institute, 2016).

The test locations are the submitting clinic’s postcodes and not the dog owner’s home postcode. Submissions were largely from practices in urban areas which may have led to a false increase in the importance of urban/suburban areas for leptospira transmission. Clients will sometimes travel varying distances to seek veterinary treatment, which might have impacted our ability to assess for highly localised risk factors. However, climate and livestock variables were explored using 5 km^2^ resolution and submissions classified by region to minimise this impact to some extent. Other studies have used IDEXX leptospirosis test submissions (in America) and therefore used location information at the clinic postcode level only (Lee et al., 2014; Smith et al., 2019; White et al., 2017). Furthermore, given that the onset of clinical signs from infection ranges between 7–14 days, dogs are likely to be walked across a wide range of environments, so therefore even home postcode data would not be ideal for determination of localised risk factors without being able to collect further information on location of walks and potential risk factor activities Ghneim et al. (2007).

The diagnosis of leptospirosis remains a challenge especially as result interpretation and sample timing remains a limitation in the diagnosis of leptospirosis across all species (Limmathurotsakul et al., 2012; Picardeau, 2013). MAT titres can be challenging to interpret, in light of potential vaccine antibody intereference and sampling early on in infection prior to seroconversion. Single MAT results have low sensitivity, this increases when paired sample are taken but this is rarely performed in primary-care practice. PCR diagnosis is best suited to early in infection and prior to antimicrobial therapy and the sensitivity and specificity varies according to target gene (Miotto et al., 2018; Schuller et al., 2015). Unfortunately, in this submissions dataset we do not have dog vaccine status, clinical history or information about whether a sample is single or paired.

One of the main challenges associated with modelling the spatial distribution of disease is identifying suitable spatial data or proxies, and understanding the uncertainties introduced as a result of the variabilities associated with the data.Previous studies have reported links between leptospirosis cases and flooding but this was not seen here (Dechet et al., 2012; Lau et al., 2010; Smith et al., 2013) Furthermore, due to open access data limitations our flooding variable was based on fluvial (river) flood hazard rather than pluvial (rainfall or surface water) flooding. Surface area flooding may be associated with canine leptospirosis more than fluvial flooding because urban areas are more susceptible to it, but this is not well understood (Houston et al., 2011). The methodologies used to determine dog density were less comprehensive than those used for livestock density calculations. Livestock densities were calculated using government sources such as mandatory animal tracing schemes. Dog density was estimated through a combination of the UK pet population using estimations based on extrapolation of survey results to UK household census data, veterinary practice locations and a clinic catchment area based on number of veterinarians and road access (Aegerter et al., 2017; Gilbert et al., 2018; Murray et al., 2010). Since leptospires have numerous wildlife reservoirs, with rodents being most notable, exploring the relationship between environment suitability and rodent density would have been of great interest (Adler, 2015), although no meaningful dataset of rodent density across GB was available.

## 5 Conclusions

This study has indicated that canine leptospirosis may exhibit a seasonal pattern in GB, with the disease increasing in Autumn. Spatial variation in both the distribution of test-positive proportions and submissions was identified, potentially indicating that agroecological risk factors and varying awareness of the disease impact the distribution of the leptospirosis in GB. Ecological niche modelling identified average annual temperature as the most important variable in all the Maxent models, accounting for between 35.9–58.5% of the variation in distribution of all cases and individual serogroups, indicating a higher probability for the presence of leptospirosis in southern and central England. However, the serogroups displayed distinct ecological preferences, most notably that Sejroe had a preference for lower average annual temperatures and that the presence of Australis was associated with areas of higher horse density. Existing vaccines provide protection against two of the three main serogroups identified here but are unlikely to provide protection against the Sejroe serogroup. Although the test-positive proportion was low in Scotland, the predominant serogroup identified in Scotland was Sejroe. Leptospirosis should still be considered as a differential diagnosis in dogs with appropriate clinical signs, even if vaccinated with a commercial tetravalent vaccine.

## Availability of data and material

The datasets generated and analysed during the current study are not publicly available due to their use in ongoing primary research, but subsections may be made available from the corresponding author on reasonable request.

## Funding

This work was undertaken as part of a PhD studentship as part of the LIDo programme and was jointly funded by the 10.13039/501100000268BBSRC [grant number BB/M009513/1] and MSD Animal Health. The funding sources had no involvement in study design, data collection, analyses or decision to publish.

## Declaration of Competing Interest

The authors report no declarations of interest.
